# Canalization reduces the nonlinearity of regulation in biological networks

**DOI:** 10.1038/s41540-024-00392-y

**Published:** 2024-06-13

**Authors:** Claus Kadelka, David Murrugarra

**Affiliations:** 1 Department of Mathematics, Iowa State University, 411 Morrill Rd, Ames, 50011 IA USA; 2 Department of Mathematics, University of Kentucky, 719 Patterson Office Tower, Lexington, 40506 KY USA

**Keywords:** Nonlinear dynamics, Applied mathematics

## Abstract

Biological networks, such as gene regulatory networks, possess desirable properties. They are more robust and controllable than random networks. This motivates the search for structural and dynamical features that evolution has incorporated into biological networks. A recent meta-analysis of published, expert-curated Boolean biological network models has revealed several such features, often referred to as design principles. Among others, the biological networks are enriched for certain recurring network motifs, the dynamic update rules are more redundant, more biased, and more canalizing than expected, and the dynamics of biological networks are better approximable by linear and lower-order approximations than those of comparable random networks. Since most of these features are interrelated, it is paramount to disentangle cause and effect, that is, to understand which features evolution actively selects for, and thus truly constitute evolutionary design principles. Here, we compare published Boolean biological network models with different ensembles of null models and show that the abundance of canalization in biological networks can almost completely explain their recently postulated high approximability. Moreover, an analysis of random N–K Kauffman models reveals a strong dependence of approximability on the dynamical robustness of a network.

## Introduction

Biological systems are frequently represented as networks, which describe the interactions between different biological entities such as genes, proteins, or metabolites. For instance, gene regulatory networks (GRNs) describe how a collection of genes governs key processes within a cell. A *static* biological network is completely described by a wiring diagram, which contains nodes (e.g., genes) and edges between nodes, which can be undirected (e.g., in protein–protein interaction networks), directed, and even signed (e.g., in gene regulatory networks). Static networks are, however, insufficient to obtain accurate insights into the often complex, non-linear dynamics of biological networks^1^. *Dynamic* biological networks possess additional information on how each node is regulated by the set of regulators. Popular dynamic modeling frameworks include differential equation models and discrete models. While the former harbors the potential for quantitative predictions, it requires a substantial amount of data for accurate inference of its many kinetic parameters. Therefore, many modelers prefer discrete models and their qualitative predictions. Boolean networks constitute the simplest type of discrete model. Here, each node takes on only two values, and time is discretized as well. The two values can be interpreted as low and high concentration, unexpressed and expressed genes or proteins, etc. Particularly for GRNs, Boolean networks have become increasingly popular. Over 160 Boolean GRN models have been curated by experts in their respective fields - most over the course of the last twelve years^2^. These models range in size from 3 to 302 nodes and describe various processes in many species and kingdoms of life.

Over the last few decades, a number of interesting features of biological networks have been identified. At the structural “wiring diagram" level, biological networks are sparsely connected with an average degree of about 2.5 and are enriched for certain network motifs such as coherent feed-forward loops and complex feedback loops, particularly those that contain many negative interactions^2,3^. Dynamically, most biological networks operate at the critical edge between order and chaos^2,4,5^. For random *N* − *K* Kauffman networks, it is well-established that the network dynamics are generally ordered whenever 2*Kp*(1 − *p*) < 1 and chaotic whenever 2*Kp*(1 − *p*) > 1; at 2*Kp*(1 − *p*) = 1, a phase transition happens^6,7^. Here, *K* is the average degree of the network, while *p* describes the *bias* of picking one in the Boolean function’s truth table; the unbiased case corresponds to *p* = 0.5, and the *absolute bias* can be quantified by 2∣0.5 − *p*∣ ∈ [0, 1], or alternatively by 1 − 4*p*(1 − *p*) ∈ [0, 1], with 0 corresponding in both cases to the unbiased case. Networks with ordered dynamics typically possess few and short attractors, while chaotic dynamics are characterized by the presence of many long attractors^8^.

The dynamic update rules of Boolean biological network models are also remarkable. They are highly canalizing, redundant, and have a high absolute bias^2,9,10^. *Canalization* is a widely used term in biology. First coined by developmental geneticist Waddington in the 1940s^11^, it refers to the tendency of developmental processes to follow particular trajectories, despite internal and external perturbations^12^. In other words, it refers to low variation in phenotypes despite potentially high variation in genotypes and the environment^13^. Correspondingly, Kauffman introduced Boolean *canalizing* functions as suitable update rules to describe the gene regulatory logic^14^. A canalizing function possesses a canalizing variable, which, when it receives its canalizing input, determines the output of the function, irrespective of all other inputs. If the subfunction which is evaluated when the canalizing variable does not receive its canalizing input is also canalizing, the function is 2-canalizing, etc.^15^. If all *n* variables of a function become eventually canalizing, the function is *n*-canalizing, also known as *nested canalizing*^16^. The number of variables that become eventually canalizing is known as the *canalizing depth*^15^. Every non-zero Boolean function possesses a unique standard monomial form, from which the canalizing depth and the number of variables in each “layer” of canalization can be directly derived^17,18^. As the number of variables increases, canalizing and especially nested canalizing functions become increasingly rare^19–21^. It is, therefore, very surprising that almost all rules in published Boolean biological network models are canalizing and even nested canalizing^2,9^.

Another recently discovered feature of biological Boolean network models is the high *approximability* of their dynamics by linear and low-order continuous Taylor approximations of the Boolean update rules^22^. Here, the mean approximation error (MAE) is defined as follows: each update rule of a given Boolean network is replaced by a continuous Taylor approximation of a defined order. The MAE describes the mean squared error between the long-term state of the Boolean network and the long-term state of the continuous approximation when starting from a random initial state (see “Methods” for details). Manicka et al found that biological networks were consistently more approximable (i.e., had lower MAE values) than random networks with the same wiring diagram (i.e., matching degree distribution) and matching update rule bias^22^.

Many of the described remarkable features of biological networks are interrelated and correlated. For instance, canalizing Boolean functions are, on average, more redundant and have a higher absolute bias than random functions^2^. In this paper, we show that the described increased approximability of biological networks can be almost fully explained by the abundance of canalization, which was not considered in^22^. We further show that the approximability of a Boolean network depends mostly on its dynamic regime, which in turn depends on its update rules (that is, average degree, bias, and amount of canalization)^2,23^. A network with ordered dynamics (i.e., few and short attractors) tends to possess much more approximable dynamics than a network with chaotic dynamics. For questions related to the interpretation of approximability from a biological perspective (e.g., what it means for a biological network to be highly approximable), we refer the interested reader to^22^.

## Results

**Fig. 1:**
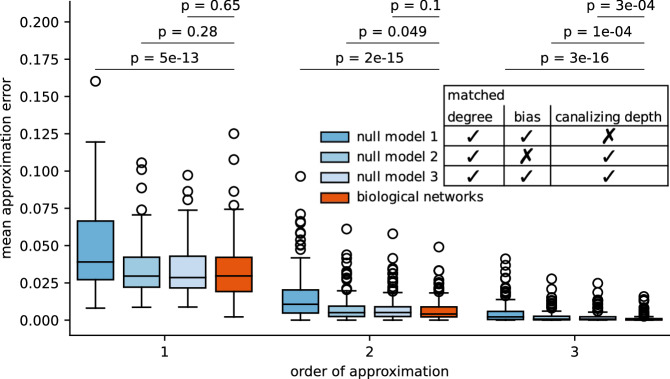
Canalization explains the high approximability of biological networks. The distribution of mean approximation errors is shown for the biological networks (orange) and three different types of random null networks (shades of blue), which match different characteristics (bias and/or canalizing depth) of the biological network. Each box depicts the interquartile range (IQR), each whisker extends to the most extreme value within 1.5 IQR from the box, and each horizontal line within a box depicts the median. For a fixed approximation order (1–3, *x*-axis), differences between the MAE distribution of the biological and the random networks are assessed using the two-sided Wilcoxon signed-rank test. Figure 2 contains scatterplots showing the MAE values of all biological networks and their random null models.

**Fig. 2:**
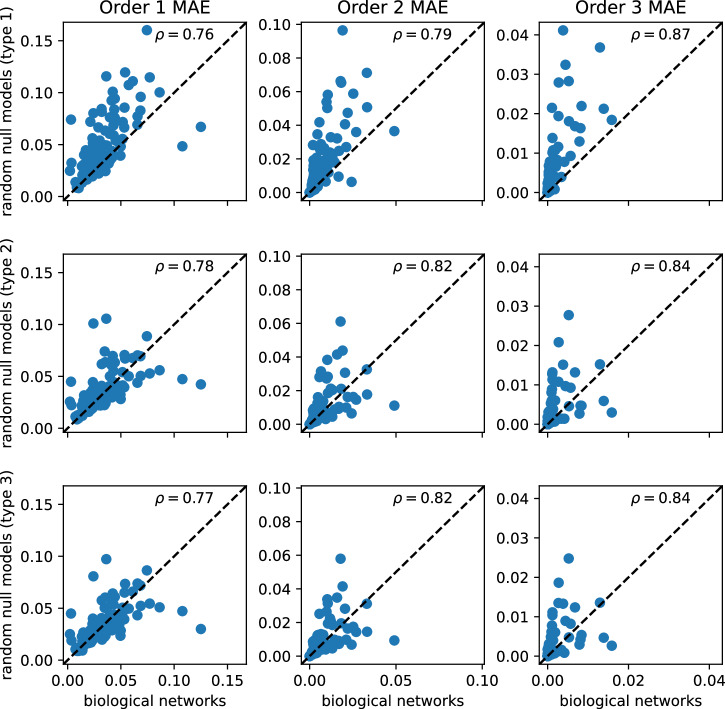
Mean approximation errors of biological networks and their random null models. For a fixed approximation order (1–3, columns), differences between the MAE values of the 110 biological and the different random networks (rows) are shown, in addition to the Spearman correlation coefficient, *ρ*. A summary of this data is shown in Fig. 1.

To test the hypothesis that the increased canalization in biological networks explains their increased approximability, we compared the approximability of published expert-curated biological networks with several ensembles of random null models, similar to^22^. All random networks possessed the same wiring diagram as the respective biological network. The authors in^22^ considered an “unconstrained” null model, where each biological update rule was replaced by a non-constant random Boolean function (of the same degree), and a “constrained" model (null model type 1 in this study), which additionally matched the bias of each biological update rule. Neither model accounted for the high degree of canalization in biological networks. We therefore considered two additional null models, one which matches the degree and canalizing depth of each biological update rule (null model type 2), and one which matches degree, canalizing depth and bias (null model type 3; see Methods for details). Note that additional null models could have been considered, even more stringent ones by matching, e.g., the exact canalizing layer structure^17^. However, such null models would potentially only possess low variation in their dynamics, complicating the interpretation of results. After excluding highly similar biological models (to avoid the introduction of selection bias) and those with a maximal degree of eleven or more (see Methods), we compared the approximability of 110 published expert-curated biological Boolean network models^2^ and the three different ensembles of null models. As in^22^, we found that random networks of type 1 were less approximable (Fig. 1). However, random networks that accounted for the increased canalization (null models of type 2 and type 3) exhibited similar levels of approximability as the biological networks. Interestingly, the higher the order of employed approximation the more significant were the differences, quantified by *p*-values from a Wilcoxon signed-rank test, in the MAE distributions between biological and random networks (Fig. 2). Third-order Taylor approximations recovered the dynamics of biological networks slightly better than those of random networks with matched degree, bias and canalizing depth.

**Fig. 3:**
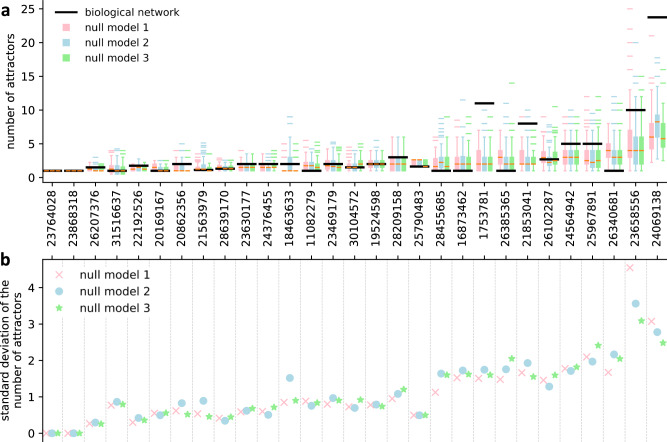
Variability in the dynamics of random null models. For all biological networks of size 15 and smaller (29 out of 110, identified by Pubmed ID) and their random null models (100 of each type), we computed the **a** average number of different network attractors per initial condition (a network with *c* external parameters has 2^*c*^ initial conditions), and **b** the standard deviation of the distribution from (**a**). Each box in **a** extends across the interquartile range (IQR), whiskers extend to the lowest data point still within 1.5 IQR of the lower quartile, and the highest data point still within 1.5 IQR of the upper quartile, horizontal colored lines (outside the whiskers) show outliers and the horizontal orange line (in the box) shows the median.

To ensure these findings are not simply due to a lack of variation in the dynamics of the null models, especially for the most constrained null model of type 3, we computed the variability in the number of network attractors among the null models (Fig. 3). To enable an exhaustive attractor search, we restricted this analysis to the 29 out of 110 Boolean networks with 15 or fewer nodes. We observed no significant change in the standard deviation of the number of attractors between null models of type 1, 2, and 3, indicating that the stringency of the constraints does not affect the findings shown in Figs. 1 and 2. Overall, these results show that the approximability of biological networks can be almost entirely explained by their high degree of canalization, measured by the canalizing depth.

However, a related question, which has implications for the control of Boolean networks^24^, remains: Why can the dynamics of biological networks be approximated so well by low-order and even linear continuous Taylor approximations? We hypothesized that the approximability of a Boolean network is strongly correlated with its dynamical robustness, which is typically measured by the average sensitivity^7^ and Derrida values^25,26^. That is, we thought that networks with robust dynamics are more approximable because they possess typically few and short attractors^27^. The robustness metrics describe how a small perturbation affects the network over time. If the perturbation gets on average smaller after each node has been synchronously updated once, the system operates in the *ordered* regime; if, on average, it increases in size, the system is in the *chaotic* regime, and if it remains, on average, of similar size, the system exhibits *criticality*. All biological systems that have thus far been modeled as Boolean networks operate close to the critical edge between order and chaos^2,4,5^. This is likely because most update rules in biological networks are nested canalizing - in fact, biological networks are even particularly enriched for insensitive nested canalizing functions (NCFs)^2^—and the expected average sensitivity of an NCF in any number of variables is 1. On the contrary, the average sensitivity of random Boolean functions with degree *k* and bias *p* is 2*kp*(1 − *p*). That is, it increases as the number of inputs increases and decreases as the function becomes more biased (where *p* = 0.5 corresponds to the unbiased case). Boolean networks governed by such random functions thus exhibit a phase transition at 2*kp*(1 − *p*) = 1^6,7^.

**Fig. 4:**
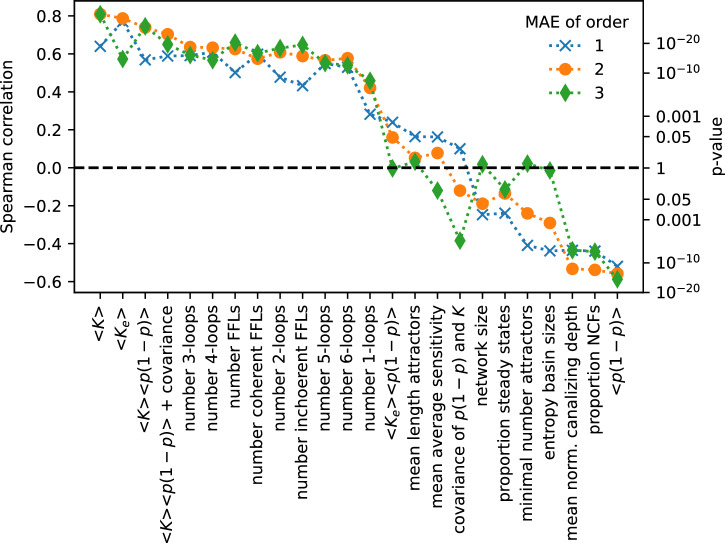
Predictors of approximability of biological networks. Pairwise Spearman correlation between the first-, second- and third-order mean approximation errors and various network properties across the 110 published biological networks, ordered by the mean correlation. 〈⋅〉 denotes the mean, *p* = output bias, *K* = number of variables, *K*_*e*_ = effective connectivity. The pairwise Spearman correlations between all shown properties are in Supplementary Fig. 1.

To test which features of a biological network make it highly approximable, we computed Spearman correlations (*ρ*) between the mean approximation errors of the 110 biological networks and several structure- and dynamics-related properties (Fig. 4). Highly connected networks proved less approximable (*ρ* > 0.6). This is likely due to the fact that a continuous Taylor approximation of order *n* matches a Boolean function with *k* ≤ *n* variables perfectly everywhere. Thus, the higher the average degree, 〈*K*〉, of a Boolean network, the lower the chance for perfect matches. Highly connected, large networks generally possess more recurring patterns, so-called network motifs. It was thus not surprising that biological networks with many feed-forward loops (FFLs) and/or feedback loops proved less approximable. Across the three approximation orders, the average degree 〈*K*〉 and the average effective degree 〈*K*_*e*_〉, defined in^10^, proved roughly equally negatively correlated with network approximability. This is somewhat surprising because the latter, which takes into account the importance of Boolean inputs, is a much stronger predictor of the dynamical robustness of a Boolean network, measured by its mean average sensitivity^2,23^. In line with this, the strongest predictor of the mean average sensitivity of a Boolean network, 〈*K*_*e*_〉〈*p*(1 − *p*)〉, as well as the mean average sensitivity itself were both not strongly correlated with the approximability of a Boolean network, with the correlation becoming insignificant for higher-order approximations. One possible explanation for this lack of strong correlation between approximability and dynamic robustness is the fact all these metrics, including the mean average sensitivity (that is, the Derrida coefficient), are ineffective measures of the true dynamic regime of a Boolean network. That is, they cannot accurately predict if two states will eventually (i.e., at time *t* = *∞*) transition to the same network attractor or not^28–30^. On the contrary, the mean normalized canalizing depth of a biological network as well as the proportion of Boolean rules, which are nested canalizing, were fairly strongly correlated with the approximability for all orders of approximation (∣*ρ*∣ > 0.4). The higher these values, the more approximable the network. Canalizing rules, especially those with a low sensitivity, have typically a fairly high absolute bias. In line with the result on the proportion of NCFs, more biased networks proved more approximable (∣*ρ*∣ > 0.5). Biological Boolean rules with a higher number of inputs tend to possess a higher absolute bias^5^. Interestingly, the covariance between *p*(1 − *p*) and the in-degree was the only property that became more correlated with approximability at higher approximation orders.

Metrics that explicitly describe dynamic aspects of a Boolean network also exhibited interesting correlations with the approximability. Assuming as in the computation of approximability^22^ a synchronous update of all nodes, we obtained, through simulation, for each biological network a lower bound of the number of attractors, as well as the approximate mean length of the attractors, the proportion of steady state attractors and the entropy of the basin sizes (see “Methods”). While the third-order approximability was not correlated with any of these metrics, networks with more attractors, a lower proportion of steady state attractors, and higher entropy possessed dynamics that were less approximable at first and second order. This goes against our hypothesis that networks with robust dynamics are highly approximable since the presence of many long attractors, and concomitant high entropy is associated with Boolean networks that operate in the chaotic regime^27^. We further lack an explanation as to why the correlation of the MAE with dynamics-related metrics generally decreases when considering higher-order approximations, while this appears not to be the case for structural metrics.

**Fig. 5:**
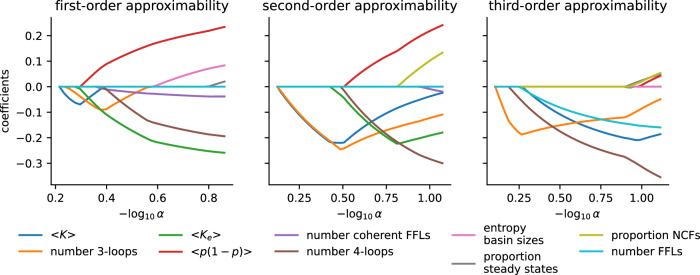
Regularization path of a linear LASSO regression to identify best predictors of approximability. A linear model with variable regularization strength (*α*) was fitted to explain the approximabilty of the 110 biological networks, using all structure- and dynamics-based network properties from Fig. 4 as potential explanatory variables. For each analysis, the regularization parameter *α* is decreased until the ninth predictor with a non-zero coefficient appears.

Given the apparent correlation between many of these structure- and dynamics-related network properties (Supplementary Fig. 1), we employed a linear LASSO regression^31^ with variable regularization strength to identify the most important predictors of first-, second-, and third-order approximability of the biological networks (Fig. 5). First-order and second-order approximability proved well-explained by a linear model involving mean absolute bias, average effective degree or average degree and the number of 3- or 4-loops. Interestingly, the best predictors of third-order approximability included a number of network motif counts (number of 3- and 4-loops as well as the number of coherent FFLs). We lack a hypothesis that may serve as an explanation for this finding, beyond the trivial observation that the signal-to-noise ratio in the distribution of order 3 MAE values is substantially lower than in those for order 1 and order 2 MAE values (Fig. 2).

**Fig. 6:**
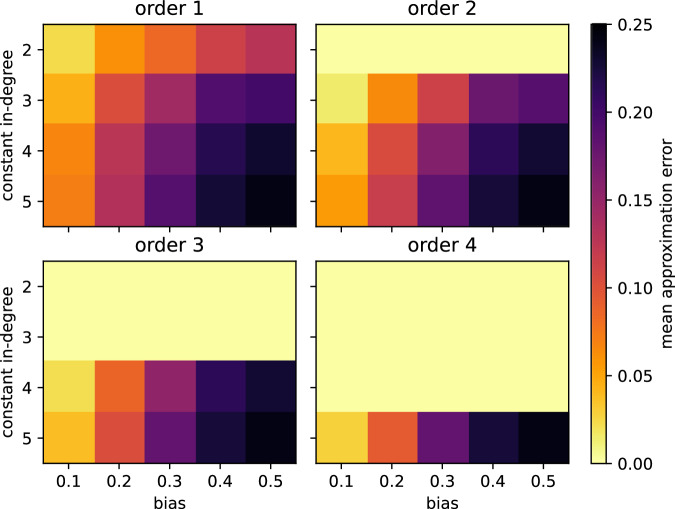
Effect of bias and in-degree on the approximability of the dynamics of Boolean networks. For strongly connected 15-node Boolean networks with a constant in-degree (*y*-axis) governed by random update functions generated with a certain bias (*x*-axis), the mean error is shown when approximating their dynamics using different order Taylor polynomials (subplots). Each cell depicts the MAE across 100 networks, and the same networks were used to estimate the MAE using first-order to fourth-order Taylor polynomials. Results from an equivalent analysis where the functions are required to be essential in all its variables are shown in Supplementary Fig. 2.

**Fig. 7:**
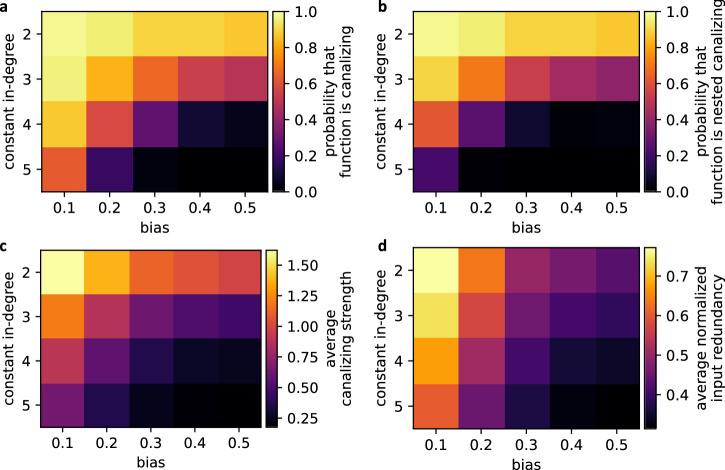
Average canalization of Boolean functions with specific bias and degree. For each combination of degree (*y*-axis) and bias (*x*-axis), 1000 random Boolean functions were generated to compute different measures of canalization: **a** probability that the function is canalizing, i.e., contains at least one canalizing variable. Note that we identify constant functions as canalizing here. **b** Probability that the function is nested canalizing, **c** average canalizing strength, as defined in^34^, **d** average normalized input redundancy, as defined in^10^.

To rule out potential confounders such as differences in network size, average degree as well as degree distribution, we considered modified N–K Kauffman networks, first defined in^32^. In these random networks of size *N*, each node has a constant degree *K*. The Boolean update rule of each node is generated by drawing 2^*K*^ times randomly with replacement from {0, 1} with probability 1 − *p* and bias *p*, respectively. We further required the wiring diagram of each network to be strongly connected since the dynamics decouple otherwise^33^. Networks with a higher absolute bias exhibited more approximable dynamics (Fig. 6). Moreover, sparse networks (i.e., with low in-degree) were, on average, more approximable. Interestingly, the MAE did not always decrease as the approximation order increased. For unbiased networks with high in-degree (e.g., *K* = 5, *p* = 0.5), the MAE was very close to the maximally observed value of 0.25, even when using fourth-order Taylor approximations. Low-degree functions with a high absolute bias exhibit the highest degree of canalization, irrespective of whether canalization is measured on the variable level^14,16^ or the function level^10,34^ (Fig. 7). The amount of canalization in N–K Kauffman networks correlates thus highly with their approximability.

Since a Boolean function with *K* inputs is perfectly matched everywhere by a continuous Taylor approximation of order *K*, the MAE values were zero in these cases. If only *J* < *K* of the inputs of a Boolean function are essential, then the *J*th order Taylor approximation already provides a perfect match. Note that a Boolean input is non-essential if a change in this input never changes the output of the function. For example, *f*(*x*, *y*) = *x* has a non-essential input *y*. To rule out a potentially confounding effect created by perfect matches, we required, in a sensitivity analysis, all update rules to be non-degenerated, i.e., to contain only essential variables (Supplementary Fig. 2). Most MAE values were slightly higher, likely due to the higher effective degree. Qualitatively, the results were, however, very similar.

**Fig. 8:**
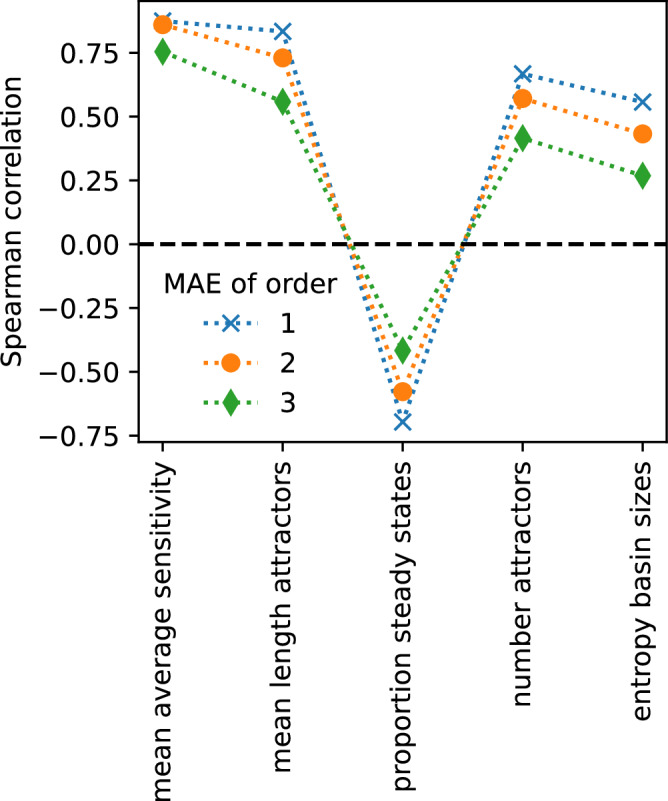
Predictors of approximability of random networks. Pairwise Spearman correlation between the first-, second-, and third-order mean approximation errors and network properties related explicitly to dynamics, across 2000 random strongly-connected Boolean networks with fixed degree *K* ∈ {2, 3, 4, 5} and bias *p* ∈ {0.1, 0.2, 0.3, 0.4, 0.5} (100 for each combination).

Combining all 2000 random networks (100 each for combinations of constant in-degree *K* ∈ {2, 3, 4, 5} and bias *p* ∈ {0.1, 0.2, 0.3, 0.4, 0.5}), we computed, as before, the Spearman correlation between MAE values and metrics that explicitly describe network dynamics. The dynamical robustness of a network, measured by the mean average sensitivity, was strongly positively correlated with first-, second-, and third-order MAE values (*ρ* > 0.75; Fig. 8). Given that the average sensitivity of random Kauffman networks is 2*Kp*(1 − *p*)^7^, this agrees qualitatively with the results from Fig. 6. Also in line is the finding that random networks are more approximable if they have few and short attractors, a high proportion of steady states, and low entropy in the distribution of the basin sizes. These four properties characterize networks that operate mostly in the ordered and critical dynamical regime. As observed for the biological networks, the correlations were consistently weaker when considering higher-order approximations. Note, however, that by design of the computational experiment, 25% (50%) of the networks perfectly match their second-order (third-order) approximation, which certainly contributed to weaker correlations.

**Fig. 9:**
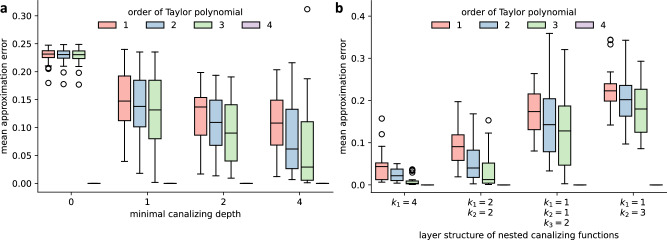
The approximability of Boolean network dynamics depends on canalization. Each boxplot shows the distribution of the mean approximation error for 100 strongly connected *N* = 15-node Boolean networks with a fixed in-degree of *K* = 4 and a variable degree of canalization, characterized by **a** the minimal canalizing depth of each update rule (*x*-axis). In **b**, all functions are nested canalizing (i.e., have canalizing depth 4) but the canalizing layer structure differs. The order of the Taylor polynomial used for the approximation is depicted by color. Fourth-order Taylor polynomials match the functions perfectly, the mean approximation error is 0. Each box extends across the interquartile range (IQR), whiskers extend to the lowest data point still within 1.5 IQR of the lower quartile, and the highest data point still within 1.5 IQR of the upper quartile, black circles show outliers and the horizontal black line shows the median.

To study the effect of canalization on the nonlinearity of regulation in more detail, we modified the random networks such that the update rules were restricted to specific classes of functions. First, we compared the approximability of random networks governed by 4-variable functions with different minimal canalizing depths (see Methods). While networks without required canalization were hardly approximable (MAE ≈ 0.25), the restriction to canalizing update rules gave rise to more approximable dynamics (Fig. 9a). Canalizing networks became increasingly more approximable as the order of the Taylor approximations increased. On the other hand, networks governed by arbitrary 4-input functions were not better approximated by higher-order Taylor approximations, unless obviously perfect matches were used. We note that functions with a higher canalizing depth are, however, on average, also less sensitive^35^ and exhibit a higher absolute bias (Fig. 7, Supplementary Fig. 1).

While the canalizing depth provides a crude measure of the amount of canalization in a Boolean function, more detailed information is contained in the canalizing layer structure^17,18,35^. To investigate this, we compared the approximability of random networks, each governed entirely by 4-variable NCFs but with different layer structure. Networks governed by NCFs with layer structure *k*_1_ = 4, e.g., an AND-NOT function ${x}_{1}\wedge {\bar{x}}_{2}\wedge {\bar{x}}_{3}\wedge {x}_{4}$, are highly approximable (Fig. 9b). On the other hand, networks governed by NCFs with layer structure *k*_1_ = 1, *k*_2_ = 3, e.g., functions such as *x*_1_ ∨ (*x*_2_ ∧ *x*_3_ ∧ *x*_4_), are much less approximable. Again, as the approximability of these networks decreases, the sensitivity of the underlying NCFs increases, and the absolute bias decreases^35^.

## Discussion

The idea of a probabilistic generalization of Boolean logic dates back all the way to George Boole^36^. In this manuscript, we study in depth a recent implementation of this idea: using continuous Taylor approximations of Boolean functions to approximate the dynamics of a Boolean network. We show that the high approximability of biological networks first postulated in^22^, can be almost entirely explained by the abundance of canalization in biological networks. We conjecture that the remaining higher approximability of biological networks is due to the reported increased occurrence of insensitive canalizing rules in biological networks^2^. Through a computational analysis of random networks, we show that the dynamical robustness of a network strongly influences its approximability: Networks with low mean average sensitivity, operating in the ordered and critical dynamical regime and characterized by few and short attractors, possess generally more approximable dynamics. In line with this, networks governed by canalizing or even nested canalizing functions, which possess a high absolute bias and are insensitive to perturbations, proved more approximable. These two findings match because such canalizing networks are known to give rise to particularly ordered dynamics with few and short attractors, mainly steady states^35,37,38^.

This study possesses a number of limitations. First, we analyze published expert-curated biological Boolean network models. Since any human is biased, the curated models are affected by bias as well. A shared bias among many modelers may give rise to abundant features in these models that are not due to evolutionary or biological reasons but purely due to this bias. In general, we cannot rule this out unless our understanding of the biology underlying these network models improves dramatically. Second, the approximability of Boolean networks, quantified by the mean approximation error and introduced in^22^, has potential shortcomings as well. It fails to consider the fact that two states may eventually transition to the same attractor but be time-shifted. Repeating the analyses in this study with a future version of approximability that considers these two states as dynamically equal, similar to the phenotypical robustness, defined in^33^, or the quasicoherence, defined in^28^, would be very interesting. However, the fact that the approximability compares the dynamics of discrete and continuous models will likely complicate this endeavor.

Assuming biological Boolean network models are worth investigating despite their biases, fully disentangling the relative contribution of the related properties canalization, bias, and sensitivity on approximability (see e.g., Fig. 9b) constitutes one of several open questions. Moreover, it remains to be investigated how well non-perfect continuous approximations of Boolean networks perform in the context of predicting control targets or specific dynamical features. A more technical question is whether Boolean functions that can be well approximated by low-order continuous extensions give rise to more approximable Boolean networks.

## Methods

### Boolean networks

A *Boolean network* *F* in variables *x*_1_, …, *x*_*n*_ can be viewed as a function on binary strings of length *n*, which can be described coordinate-wise by *n* *Boolean update functions* *f*_*i*_: {0, 1}^*n*^ → {0, 1}. Every Boolean network defines a canonical map, where the functions are synchronously updated:

1 $$F:{\{0,1\}}^{n}\to {\{0,1\}}^{n},\,F({x}_{1},\ldots ,{x}_{n})=({f}_{1}(x),\ldots ,{f}_{n}(x)).$$

In this paper, we only consider this canonical map, i.e., we only consider *synchronously updated Boolean networks*.

While possible, most update functions in a Boolean network do not depend on all *n* variables. The *wiring diagram* describes the dependencies. It contains *n* nodes, corresponding to the *x*_*i*_, and a directed edge from *x*_*i*_ to *x*_*j*_ if *f*_*j*_ depends on *x*_*i*_ (that is, if *f*_*j*_(*x*_1_, …, *x*_*i*_ = 0, …, *x*_*n*_) ≠ *f*_*j*_(*x*_1_, …, *x*_*i*_ = 1, …, *x*_*n*_) for at least some (*x*_1_, …, *x*_*i*−1_, *x*_*i*+1_, …, *x*_*n*_) ∈ {0, 1}^*n*−1^). If *f*_*j*_ depends on *x*_*i*_, *x*_*i*_ is a *essential* variable. Otherwise, it is *non-essential*. From the wiring diagram, the degree of each node can be derived.

### Metrics describing Boolean network dynamics

A second graph associated with a synchronously updated Boolean network *F*, the *state space*, contains as nodes the 2^*n*^ binary strings and a directed edge from *x* ∈ {0, 1}^*n*^ to *y* ∈ {0, 1}^*n*^ if *F*(*x*) = *y*. Each connected component of the state space corresponds to a basin of attraction, consisting of a directed loop, the *attractor*, as well as trees feeding into the attractor. Attractors can be steady states (also known as fixed points) or limit cycles. Due to its finite size, all states in a Boolean network eventually transition to an attractor. Every attractor in a biological network model typically corresponds to a distinct phenotype^39^.

Since the number of nodes, *n*, in the investigated biological Boolean network models differs from 3 to 302, some of the state spaces are huge (size 2^*n*^). We therefore used the following procedure to approximate several dynamics-related metrics. For each biological network *F*, we randomly picked 1000 different initial values *x*_0_ ∈ {0, 1}^*n*^. For each *x*_0_, we synchronously updated *F* until a repeated state was reached, indicating the arrival at an attractor. The number of updates between first and second transition to the repeated state corresponds to the length of the attractor. This process yields a non-empty list of attractors {*A*_1_, …, *A*_*s*_} of length {*L*_1_, …, *L*_*s*_} with corresponding basin sizes {*B*_1_, …, *B*_*s*_}. We used $\frac{1}{s}{\sum }_{i}{L}_{i}$ as the approximate mean length of the attractor and $\frac{1}{s}{\sum }_{i}1({L}_{i}=1)$ as the approximate proportion of steady state attractors. We considered an alternative version of these two measures, weighted by the relative basin sizes (that is, $\frac{1}{1000}{\sum }_{i}{B}_{i}{L}_{i}$ and $\frac{1}{1000}{\sum }_{i}{B}_{i}1({L}_{i}=1)$). Since the alternative versions differed barely from the respective base versions (Spearman correlations of *ρ* > 0.95 across the 110 investigated biological networks), we decided to only use the base versions in the analysis. We approximated the entropy of the basin sizes as $-\frac{1}{1000}{\sum }_{i}\ln (\frac{{B}_{i}}{1000}){B}_{i}\in [0,\infty )$. Note that to ensure we use the same method, we approximated the state space even for networks with small state space.

Finally, we used *s* as the lower bound of the number of attractors. In a network with many attractors, we almost certainly fail to discover all attractors when starting from only 1000 random states. However, all attractors with a large basin size are discovered with high probability.

For the random Boolean networks of fixed size *n* = 15, analyzed in Figs. 6, 8, 9 and Supplementary Fig. 2, we computed the entire state space. All dynamics-related metrics, including the number of network attractors, are therefore exact in these analyses.

### Continuous extensions of Boolean functions

To compute the approximability of Boolean networks, we use the same approach as in^22^. We start by defining continuous extensions of Boolean functions. Any Boolean function *f*: {0, 1}^*n*^ → {0, 1} is defined in the corners of the *n*-dimensional hypercube, {0, 1}^*n*^, and can be extended to the entire hypercube [0, 1]^*n*^ by defining a function $\hat{f}:{[0,1]}^{n}\to [0,1]$ such that $\hat{f}(x)=f(x)$ for all *x* ∈ {0, 1}^*n*^. Specifically, we employ a probabilistic generalization of Boolean logic, already introduced by George Booole^36^. We consider random variables *X*_*i*_: {0, 1} → [0, 1] with Bernoulli distributions and set *p*_*i*_ = Prob(*X*_*i*_ = 1). Let *X* = *X*_1_ × ⋯ × *X*_*n*_ be the product of random variables. Then, we define

2 $$\hat{f}({p}_{1},\ldots ,{p}_{n})=\sum\limits_{\begin{array}{c}x\in X:\\ f(x)=1\end{array}}\prod\limits_{i=1}^{n}{\hat{p}}_{i}$$

where

3 $${\hat{p}}_{i}=\left\{\begin{array}{ll}{p}_{i}\quad &\,{{\mbox{if}}}\,\,{x}_{i}=1,\\ 1-{p}_{i}\quad &\,{{\mbox{if}}}\,\,{x}_{i}=0.\end{array}\right.$$

By this definition, $\hat{f}:{[0,1]}^{n}\to [0,1]$ is a continuous function that satisfies $\hat{f}(x)=f(x)$ for all *x* ∈ {0, 1}^*n*^^22^.

### Taylor polynomials of Boolean functions

Since $\hat{f}$ is a continuous-variable function, we can consider different orders of approximation for $\hat{f}$ using its Taylor expansion. As described in^22^, $\hat{f}$ is a square-free polynomial and its Taylor expansion is finite. More specifically, the *n*th order approximation will match $\hat{f}$ perfectly, and if only *m* < *n* inputs of *f* are essential, then the *m*th order approximation already matches $\hat{f}$ perfectly.

For a given *α* = (*α*_1_, …, *α*_*n*_) ∈ {0, 1}^*n*^ and *x* ∈ [0, 1]^*n*^, we define

4 $$| \alpha | ={\alpha }_{1}+\cdots +{\alpha }_{n},$$

5 $${x}^{\alpha }={x}_{1}^{{\alpha }_{1}}{x}_{2}^{{\alpha }_{2}}\cdots {x}_{n}^{{\alpha }_{n}},$$

6 $${\partial }^{\alpha }\hat{f}={\partial }_{1}^{{\alpha }_{1}}{\partial }_{2}^{{\alpha }_{2}}\cdots {\partial }_{n}^{{\alpha }_{n}}\hat{f}=\frac{{\partial }^{| \alpha | }\hat{f}}{{\partial }_{1}^{{\alpha }_{1}}{\partial }_{2}^{{\alpha }_{2}}\cdots {\partial }_{n}^{{\alpha }_{n}}},$$

with the convention that ${\partial }_{i}^{0}\hat{f}\equiv \hat{f}$. For *p* ∈ [0, 1]^*n*^, we have

7 $$\hat{f}(x)=\sum\limits_{\alpha \in {\{0,1\}}^{n}}\frac{{\partial }^{\alpha }\hat{f}(p)}{| \alpha | !}{(x-p)}^{\alpha }=\hat{f}(p)+\sum\limits_{\begin{array}{c}\alpha \in {\{0,1\}}^{n}\\ 0 < | \alpha | \le n\end{array}}\frac{{\partial }^{\alpha }\hat{f}(p)}{| \alpha | !}{(x-p)}^{\alpha }.$$

If $p=(\frac{1}{2},\ldots ,\frac{1}{2})$, which represents the unbiased selection of each variable, then $\hat{f}(p)$ equals the output bias of *f*, as shown in^22^. The Taylor decomposition yields different approximations of a Boolean function by restricting the sum in Eq. (7) to *α* with ∣*α*∣ ≤ *m* ≤ *n*. The Taylor polynomial of order *m* is given by

8 $${\hat{f}}^{(m)}(x)=\sum\limits_{\begin{array}{c}\alpha \in {\{0,1\}}^{n}\\ | \alpha | \le m\end{array}}\frac{{\partial }^{\alpha }\hat{f}(p)}{| \alpha | !}{(x-p)}^{\alpha }$$

### Approximability of a Boolean network by continuous extensions

Let *F* = (*f*_1_, ⋯ , *f*_*n*_): {0, 1}^*n*^ → {0, 1}^*n*^ be a Boolean network. We define the *m*th order approximation of *F* to be

9 $${\hat{F}}^{(m)} =\left(\max\left(0,\min\left(1,{\hat{f}}_1^{(m)}\right)\right),\ldots,\max\left(0,\min\left(1,{\hat{f}}_n^{(m)}\right)\right)\right):[0,1]^n\to[0,1]^n,$$

where the update functions of ${\hat{F}}^{(m)}$ are the *m*th order Taylor approximations of the update functions of *F*, ${\hat{f}}_{i}^{(m)}$ as defined in Equation (8), rescaled to the interval [0, 1].

With this, we can define the mean approximation error (MAE) as the mean squared error between the long-term state of the Boolean network and the long-term state of its continuous approximation. That is,

10 $$\,{{\mbox{MAE}}}\,(F,m)=\frac{1}{{2}^{n}}\sum\limits_{{x}_{0}\in {\{0,1\}}^{n}}\parallel {F}^{\infty }({x}_{0})-{\hat{F}}^{(m),\infty }({x}_{0}){\parallel }^{2}$$

where *F*^*∞*^(*x*_0_) and ${\hat{F}}^{(m),\infty }({x}_{0})$ describe the long-term state of the Boolean network *F* and its *m*th order approximation, respectively. In practice, we approximated the MAE, using the Python library boolion^22^, by updating both *F* and ${\hat{F}}^{(m)}$ synchronously 25 times and using 1000 random initial values *X* ⊂ {0, 1}^*n*^. That is, we approximate *MAE*(*F*, *m*) by computing

11 $$\frac{1}{1000}\mathop{\sum}\limits_{{x}_{0}\in X}\parallel {F}^{\infty }({x}_{0})-{\hat{F}}^{(m),\infty }({x}_{0}){\parallel }^{2}.$$

### Linear LASSO regression

To better determine the relative importance of a number of correlated structure- and dynamics-related network properties on approximability, we performed a multivariable linear LASSO regression^31^. Assume $\tilde{{{{\bf{y}}}}}$ describes the MAE of order *k* = 1, 2, or 3 for the *N* = 110 biological networks, and $\tilde{{{{{\bf{x}}}}}_{{{{\bf{i}}}}}},i=1,\ldots ,d$ describes the explanatory variables shown in Fig. 4 (*d* = 24). To enable an appropriate interpretation of the results, we first scaled each vector to have mean 0 and standard deviation 1, yielding **y** and **x**_*i*_. We then solved the following minimization problem using sklearn.linear_model in Python 3.10:

12 $$\mathop{\min }\limits_{\beta \in {{\mathbb{R}}}^{d}}\left\{\frac{1}{N}\parallel {{{\bf{y}}}}-X\beta {\parallel }_{2}^{2}+\alpha \parallel \beta {\parallel }_{1}\right\},$$

where *X* = [**x**_1_ ⋯ **x**_*d*_] is the design matrix, $\beta \in {{\mathbb{R}}}^{d}$ the vector of regression parameters, and *α* ≥ 0 the regularization (i.e., penalization) parameter. Note that the smaller *α* the larger is typically the number of explanatory variables with non-zero parameters. In the LASSO regularization paths, shown in Fig. 5, we reduced *α* until nine properties with non-zero parameter *β*_*i*_ had emerged.

### Canalization

This study employs several mathematical concepts related to canalization. By^14^, a Boolean function *f*(*x*_1_, …, *x*_*n*_): {0, 1}^*n*^ → {0, 1} is canalizing if there exists a canalizing variable *x*_*i*_, a canalizing input *a* ∈ {0, 1} and a canalized output *b* ∈ {0, 1} such that

13 $$f({x}_{1},\ldots ,{x}_{n})=\left\{\begin{array}{ll}b\quad &\,{{\mbox{if}}}\,{x}_{i}=a,\\ g({x}_{1},\ldots ,{x}_{i-1},{x}_{i+1},\ldots ,{x}_{n})\quad &\,{{\mbox{otherwise.}}}\,\end{array}\right.$$

Some authors argue that constant functions are not canalizing, thus requiring the subfunction *g* to differ from *b*^17^. If *g* is also canalizing, then *f* is 2-canalizing, etc. More generally, *f* is *k*-*canalizing*, where 1 ≤ *k* ≤ *n*, with respect to the permutation $\sigma \in {{{{\mathcal{S}}}}}_{n}$, inputs *a*_1_, …, *a*_*k*_, and outputs *b*_1_, …, *b*_*k*_ if

14 $$f({x}_{1},\ldots ,{x}_{n})=\left\{\begin{array}{ll}{b}_{1}\quad &{x}_{\sigma (1)}={a}_{1},\\ {b}_{2}\quad &{x}_{\sigma (1)}\,\ne\, {a}_{1},{x}_{\sigma (2)}={a}_{2},\\ {b}_{3}\quad &{x}_{\sigma (1)}\,\ne\, {a}_{1},{x}_{\sigma (2)}\,\ne\, {a}_{2},{x}_{\sigma (3)}={a}_{3},\\ \vdots \quad &\vdots \\ {b}_{k}\quad &{x}_{\sigma (1)}\,\ne\, {a}_{1},\ldots ,{x}_{\sigma (k-1)}\,\ne\, {a}_{k-1},{x}_{\sigma (k)}={a}_{k},\\ {f}_{C}\not\equiv {b}_{k}\quad &{x}_{\sigma (1)}\,\ne\, {a}_{1},\ldots ,{x}_{\sigma (k-1)}\,\ne\, {a}_{k-1},{x}_{\sigma (k)}\,\ne\, {a}_{k}.\end{array}\right.$$

Here, *f*_*C*_ = *f*_*C*_(*x*_*σ*(*k*+1)_, …, *x*_*σ*(*n*)_) is the *core function*, a Boolean function on *n* − *k* variables. When *f*_*C*_ is not canalizing, then the integer *k* is the *canalizing depth* of *f*^15^. If *k* = *n* (i.e., if all variables are become eventually canalizing), then *f* is a *nested canalizing function* (NCF)^16^. By^17^, every nonzero Boolean function *f*(*x*_1_, …, *x*_*n*_) can be uniquely written as

15 $$f({x}_{1},\ldots ,{x}_{n})={M}_{1}({M}_{2}(\cdots ({M}_{r-1}({M}_{r}{p}_{C}+1)+1)\cdots )+1)+q,$$

where each ${M}_{i}=\mathop{\prod }\nolimits_{j = 1}^{{k}_{i}}({x}_{{i}_{j}}+{a}_{{i}_{j}})$ is a non-constant extended monomial, *p*_*C*_ is the *core polynomial* of *f*, and $k=\mathop{\sum }\nolimits_{i = 1}^{r}{k}_{i}$ is the canalizing depth. Each *x*_*i*_ appears in exactly one of {*M*_1_, …, *M*_*r*_, *p*_*C*_}. The *layer structure* of *f* is the vector (*k*_1_, *k*_2_, …, *k*_*r*_) and describes the number of variables in each layer *M*_*i*_^18,35^.

### Published biological Boolean network models

As part of a recent meta-analysis of 122 published biological Boolean network models, a repository of 163 such models was created^2^. All 110 models analyzed in this study come from this repository. As in^2^, we excluded highly similar models to avoid the introduction of selection bias. That is, for each set of models with highly similar variables (where similarity was assessed using the Szymkiewicz-Simpson “overlap" coefficient^40^), we only kept the most final version of the model. This led to the exclusion of 39 of the 163 models. For details, see^2^. Moreover, as the MAE computation for networks with high in-degree is very computationally expensive, we only considered networks with a maximal in-degree of ten or lower. This led to the exclusion of 12 additional models, yielding a total of 110 models, which were analyzed in this study. We note that in the initial analysis of approximability of biological networks, reported in^22^, highly similar models were not excluded.

### Random null models of biological networks

We compared biological Boolean network models to three ensembles of null models that matched different characteristics of the biological networks, as shown in Fig. 1. All null models matched the in-degree of the biological networks. Null models 1 and 3 matched, in addition, the bias of each biological update rule, while null models 2 and 3 matched the canalizing depth.

Let *F* = (*f*_1_, …, *f*_*n*_) be a biological Boolean network model. For each *f*_*i*_, we first simplified the function to only include essential variables, yielding ${\tilde{f}}_{i}:{\{0,1\}}^{k}\to \{0,1\}$, where *k* is the number of essential variables, i.e., the in-degree. While this step was omitted in^22^, it appears important for an unbiased comparison given that close to 2% of regulators in biological networks are non-essential^2^. We then computed the number of ones in the truth table of ${\tilde{f}}_{i}$, denoted *q*, and ${\tilde{f}}_{i}$’s canalizing depth *d*, following^18^.

To obtain a random Boolean function *g* (for null model 1) with the same bias as ${\tilde{f}}_{i}$ and arbitrary canalizing depth, we simply selected a random subset Ω ⊆ {0, 1}^*k*^ of size ∣Ω∣ = *q*, and set

16 $$g(x)=\left\{\begin{array}{ll}1\quad &\,{{\mbox{if}}}\,\,x\in \Omega \\ 0\quad &\,{{\mbox{if}}}\,\,x\,\notin \,\Omega .\end{array}\right.$$

To obtain a random Boolean function *g* (for null model 2) with exact canalizing depth *d* and arbitrary bias, we randomly selected *d* out of ${\tilde{f}}_{i}$’s *k* essential variables, arranged them in a random order, and randomly selected for each of the *d* variables a canalizing input value *a* ∈ {0, 1} and a canalized output value *b* ∈ {0, 1} (see Equations (13), (14)). Finally, we randomly selected a core function *g*_*C*_: {0, 1}^*k*−*d*^ → {0, 1}, ensuring that *g*_*C*_ depends on all *k* − *d* variables and that *g*_*C*_ is not canalizing, by repeating this random selection process until both conditions were met. We then filled the truth table of *g*, as outlined in Equation (14). This entire procedure has already been implemented in the Python library canalizing_function_toolbox, published along with^2^.

To obtain a random Boolean function *g* (for null model 3) with the same bias as ${\tilde{f}}_{i}$ and the same canalizing depth *d*, we followed the same procedure as for null model 2, with two exceptions. First, we did not randomly select the canalized output values *b*_1_, …, *b*_*d*_ but instead used the canalized output values of ${\tilde{f}}_{i}$. Otherwise, it is impossible to obtain the same bias. Second, we randomly selected a core function *g*_*C*_ of *g* that has the same number of ones as the core function of ${\tilde{f}}_{i}$ (following the same approach as for null model 1).

Many biological networks contain external parameters, which remain constant over time. In an *n*-node Boolean network, a variable *x*_*i*_ is an external parameter if its update function is *f*_*i*_(*x*_1_, …, *x*_*n*_) = *x*_*i*_. That is, if *x*_*i*_(0) = *a* ∈ {0, 1}, then *x*_*i*_(*t*) = *a* for all *t* ≥ 0. An *n*-node Boolean network with *m* < *n* of the nodes external parameters can be seen as 2^*m*^ distinct Boolean networks since all 2^*m*^ state spaces are disconnected. Specifically, the Boolean network has at least 2^*m*^ attractors. In the null model generation, we ensured that external parameters remained external parameters. That is, we did not allow for external parameters *x*_*i*_ to obtain the *f*(*x*_1_, …, *x*_*n*_) = ¬ *x*_*i*_ even though this rule has the same degree, bias and canalizing depth.

### Random Boolean networks

To generate a random Boolean network *F* = (*f*_1_, …, *f*_*N*_) (modified *N* − *K* Kauffman network), we first generated a random directed graph of *N* nodes (the wiring diagram), where each node has *K* incoming edges. We ensured the graph is simple (i.e., does not contain self-edges/auto-regulations). We further ensured the graph is strongly connected since the dynamics decouple otherwise^33^.

To obtain the random Boolean update rules *f*_1_, …, *f*_*N*_, we randomly selected, for the networks analyzed in Figs. 6, 8, any Boolean function *g*: {0, 1}^*K*^ → {0, 1}. In a sensitivity analysis, reported in Supplementary Fig. 2, we ensured that *g* is non-degenerated, i.e., that all variables of *g* are essential, by repeating the random selection until this condition was met. For the random Boolean networks with constant degree 4 and minimal canalizing depth *d* ∈ {0, 1, 2, 4}, analyzed in Fig. 9a, we followed a very similar procedure as for null model 2 (see above), with one exception: We allowed the core function to be canalizing so that the realized canalizing depth may be larger than *d*. For the random nested canalizing Boolean networks with constant degree 4 and different layer structure, analyzed in Fig. 9b, we followed again a very similar procedure as for null model 2, with the exception that the layer structure determines the canalized output values, *b*_1_, …, *b*_4_^35^.

### Reporting summary

Further information on research design is available in the Nature Research Reporting Summary linked to this article.

### Supplementary information

Supplementary Information

Reporting Summary

## Data Availability

Kadelka et al.^2^ contains standardized update rules of the 110 investigated published, expert-curated Boolean biological network models.

## Code availability

Original code to compute the approximability of Boolean networks, published along with^22^, is available at https://gitlab.com/smanicka/boolion. Code to analyze the 110 investigated published, expert-curated Boolean biological network models, as well as the Python library canalizing_function_toolbox, published along with^2^, is available at https://github.com/ckadelka/DesignPrinciplesGeneNetworks. New code underlying the analyses described in this paper is available at https://github.com/ckadelka/ApproximabilityBooleanNetworks.
