# Supplementary material for: Canalization reduces the nonlinearity of regulation in biological networks
Source: NPJ Syst Biol Appl. 2024 Jun 13;10:67. doi: 10.1038/s41540-024-00392-y (PMC11176187; doi:10.1038/s41540-024-00392-y)
Supplement: Supplementary file 1 — Supplementary Information [file 41540_2024_392_MOESM1_ESM.pdf]

Supplementary Figures

Canalization reduces the nonlinearity of regulation in biological networks

Claus Kadelka and David Murrugarra

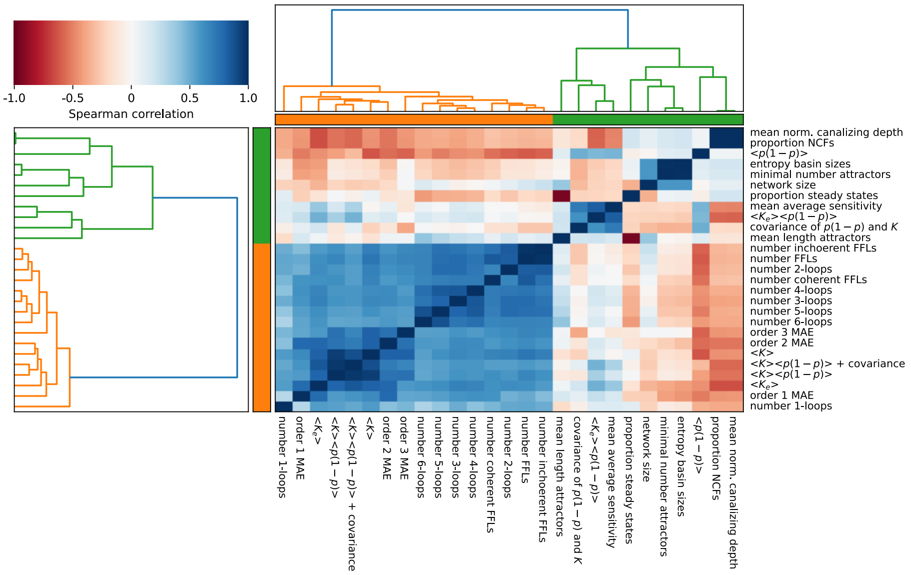

Supplementary Figure 1: Pairwise Spearman correlation between properties of the 110 published biological networks. Clusters are defined using average linkage hierarchical clustering and Euclidean distance. $< \cdot >$ denotes the mean, $p =$ output bias, $K =$ number of variables, $K_e =$ effective connectivity, covariance $=$ covariance of $p(1-p)$ and K.

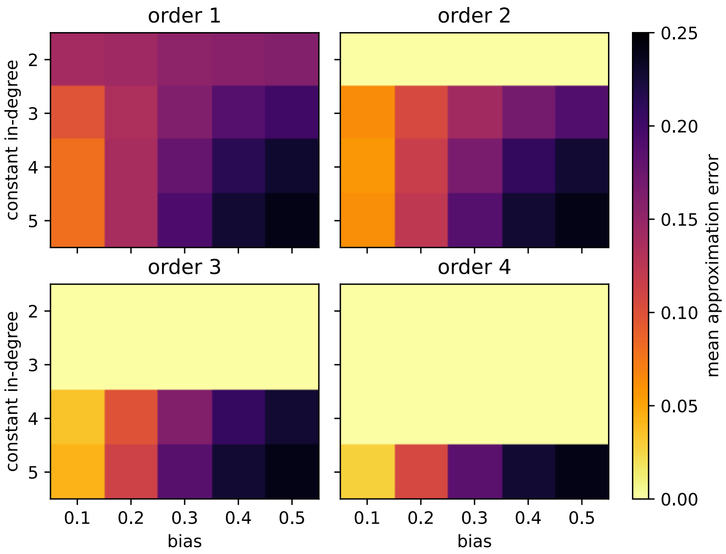

Supplementary Figure 2: **Effect of bias and in-degree on the approximability of the dynamics of non-degenerated Boolean networks.** For strongly connected 15-node Boolean networks with a constant in-degree (y-axis) governed by random non-degenerated update functions generated with a certain bias (x-axis), the mean error is shown when approximating their dynamics using different order Taylor polynomials (subplots). Each cell depicts the mean approximation error across 100 networks, and the same networks were used to estimate the mean approximation error using first-order to fourth-order Taylor polynomials. Results from an equivalent analysis where the functions are allowed to contain non-essential variables are shown in Fig. 6.
